# Supplementary material for: Myofibroblasts: A key promoter of tumorigenesis following radiofrequency tumor ablation
Source: PLoS One. 2022 Jul 20;17(7):e0266522. doi: 10.1371/journal.pone.0266522 (PMC9299299; doi:10.1371/journal.pone.0266522)
Supplement: S1 File — (DOCX) [file pone.0266522.s001.docx]

**Supplementary material and methods**

*Animal Models:* Prior to the experiment, same litter 6-8 week old female Fisher rats (F344) weighing approximately 150 g and 7–8-week-old male BALB/c and C57BL/6 mice weighing 24–31 g were purchased, housed and cared for by specially trained personnel at the respective institutions. Animals were maintained on a 12-hour light-dark cycle in a specific pathogen-free animal facility with free access to food and water. For all rat experiments and procedures, anesthesia was induced with isoflurane gas and pain alleviation was performed by administering buprenorphine-SR 300 µl (1 mg/ml) (Zoopharm, Laramie, WY) subcutaneously once immediately post-procedure. For all mouse experiments and procedures, systemic anesthesia was induced with intraperitoneal injection of a mixture of ketamine (50 mg/kg; Ketaject, Phoenix Pharmaceutical, St Joseph, Mo) and xylazine (5 mg/kg; Bayer, Shawnee Mission, Kan), and analgesic carprofen (5 mg/kg; Norbrook, Northern Ireland) was administered subcutaneously. Euthanasia for both animal models was performed by overdose of Carbon dioxide chamber system was used to sacrifice all animals (SMARTBOX CO2 chamber system; EZ Systems, Palmer, Pa).

*Tumor cell line implantation:* The model used in this experiment is a well-characterized R3230 mammary adenocarcinoma model with known and well-established tumor growth rates [35-37]. Approximately 10^7^ R3230 cells at >97% viability count and resuspended in RPMI were grown at 37°C with complete growth media (RPMI + L-glutamine + FBS and antibiotics if required) was implanted into mammary pads of female Fisher 344 rats with a mean weight 150 g ± 20 (age, 14–16 weeks) as previously demonstrated [35-37]. R3230 cells were implanted by slowly injecting 0.3 mL of R3230 cells suspension into the mammary fat pad of each animal via an 18-gauge needle. The tumor growth was measured once per day as soon as the tumor in the mammary fat pad became palpable, typically within 72 hours.

For mouse colorectal tumor implantation the spleen was exposed by a surgical peritoneal incision, whose longitudinal axis measured 5 mm in length and cut perpendicularly to that of the spleen. For this study, the colorectal adenocarcinoma cell suspension (CT26 cells for BALB/c and MC38 cells for C57BL/6 mice) was slowly implanted into each mouse over one minute via direct intraparenchymal splenic injection using a 25-gauge needle within a volume of 200ul at a concentration of 1.0*10^6^ /mL [17]. Splenectomy was performed 5 minutes post-transplantation followed by abdominal closure.

*Tumor burden and growth measurements:* R3230 breast cancer tumor growth was measured using inside calipers. The longest and shortest perpendicular diameters were recorded and used to calculate the mean diameter [35-37]. Tumors were measured daily until the days the rats were sacrificed. The animals were then randomly allocated to the various treatment groups. Serial monitoring insured at least five interval measurement points prior to ablation. The normal liver or kidney was ablated when the R3230 tumors reached 10-11 mm. The R3230 tumors were then measured once per day to obtain at least seven data points after RF ablation or sham treatment.

For the colorectal mouse model, the total number of visible tumors in each liver were quantified and stratified as being either less than or greater than 3 mm in diameter, as previously described [17]. Tumor ratios were calculated to assess overall tumor burden. The percentage of tumor area to the entire liver for each mouse was quantified. Tumor surface area was measured in pixels by tracing all the tumor borders and the whole liver surface with image processing software (ImageJ, version 1.52a, [*http://imagej.nih.gov/ij/download.html*](https://imagej.nih.gov/ij/download.html), National Institutes of Health and the Laboratory for Optical and Computational Instrumentation, University of Wisconsin, Madison, Wis) for both the ventral and dorsal sides of the liver.

*Mini-laparotomy:* After induction of anesthesia as detailed in prior sections, fur was shaved and the skin prepped with Betadine and alcohol. Using autoclaved surgical instruments; followed by bead sterilization in-between animal use, the laparotomy was performed for organ exposure and direct RF probe placement. In the case of liver RFA, under strict sterile technique a 1-2 cm right subcostal laparotomy was performed using a sharp surgical technique with a 10 or 11 scalpel. Once the peritoneum was exposed it was dissected through using a forceps and scissors. The right hepatic lobe was brought to the surface using cotton swabs. The RF probe, in rats, was inserted in normal liver tissue under direct visualization. In mouse liver RFA, to avoid piercing trauma, the RF needle was placed onto liver surface. In the case of kidney RFA, right mid laparotomy 1-2 cm was performed. The remainder of the procedure was similar to liver RF technique. Upon removal of the needle gentle pressure was applied at the exit site to achieve hemostasis. Hemostasis was confirmed through visual inspection prior to closure. The skin and peritoneum were re-approximated in a two-layer closure technique using absorbable sutures (Vicryl 4-0) for the peritoneum subcutaneous tissues and nonabsorbable sutures (Prolene 4-0) for the skin.

*RF ablation and sham procedures:* RF ablation was performed as previously described [35-37, 17], using a conventional monopolar RF ablation generator (500-kHz; model 3E; Radionics, Burlington, Mass). Briefly, a 1-cm tip of 21-gauge electrically insulated electrode was placed in the targeted (liver and kidney) area of ablation with the application of RFA to generate 70°C ± 2 for 5 min at the 1cm tip and a standardized metallic grounding pad (Radionics) to complete the circuit. Sham procedures were performed in a similar fashion without activating the RF probe.

*Drug preparation and administration:* Atorvastatin (Pfizer, Andover, MA) was dissolved in 0.9% NaCl to achieve a dose of 7.6 mg/kg for rats and 6.5mg/kg for mice. 300 µl of the drug suspension, containing the respective doses were administered intraperitoneally into the each animal. For rats treated with a single dose, administration was performed 24 hours post sham or RFA. For arms 3 daily doses were administered 15 minutes, 24 and 48 hours post RFA or sham. For mouse models the single dose treatment group was given as a single dose 3 hours prior to RFA. For the 3 dose treatment groups, doses were administered at 3 hours prior to sham or RFA procedure, 3 hours prior to intrasplenic tumor cell injection, and 24 hours after intra-splenic tumor injection.

*Tissue Harvesting:* Rats were sacrificed at 3 days or 7 days post ablation as outlined earlier and the relevant samples were harvested including blood and ablated organs. Ablated organs were bisected in a fashion perpendicular to the ablation axis. Half was flash frozen using liquid nitrogen and the other half was fixed in 10% formalin overnight then embedded in paraffin. The paraffin blocks were sliced at 5 µm thickness and stained with hematoxylin and eosin for gross pathologic examination. For the mouse model, following euthanasia, the livers were excised en bloc and photographed in toto on both the ventral and dorsal surfaces. Thereafter, livers were transected to include half of the coagulation zone including the periablational rim, tumor burden, and normal parenchyma in each specimen. Specimens were fixed in 10% formalin overnight then embedded in paraffin. The paraffin blocks were sliced at 5 µm thickness and stained with hematoxylin and eosin for gross pathologic examination.

*Immunohistochemical analysis and quantification of tumor biomarkers:* Sections from all rat harvested tissue samples were prepared and immunohistochemically stained to evaluate the effect of the adjuvant treatment on cell proliferation, microvascular density, alpha-smooth muscle actin myofibroblast, macrophages, natural killer cells, mature dendritic cells, vascular endothelial factor receptor-1 and fibroblast growth factor-19. Tissues harvested from our mouse model were evaluated for cell proliferation, microvascular density, alpha-smooth muscle actin myofibroblast, macrophages, and vascular endothelial factor receptor-1. The IHC staining was performed on 5 µm thickness paraffin. Slides were deparaffinized and hydrated. Antigen retrieval was performed in a pressure cooker by boiling the slides for 10 minutes in Tris-EDTA (pH 9) for CD80 and KIR3DL1 (NK) antibodies and 10mM sodium citrate for the remainder of the antibodies. Endogenous peroxidase activity was blocked with 3% hydrogen peroxide. After two washes each in ddH2O, TBST, and TBS, the sections were incubated with 2% Bovine Serum Albumin (ImmunoResearch) for an hour at room temperature. Slides were then incubated with α-SMA (1:500, Abcam, ab7817), CD80 (1:800, Abcam, ab215166), KIR3DL1 (NK) antibody (1:1000, Abcam, ab 233740), CD68 (1:1000, Abcam, ab31630), CD34 (1:1000, Abcam, ab81289), Ki-67 (1:200, Thermo Fisher, RM9106S), FGF-19 (1:500, Abcam, ab225942), VEGFR (1:1000, Abcam, ab32152) overnight at 4^o^C. For mouse samples, α–smooth muscle actin (Sigma Aldrich, Rehovot, Israel) for evaluation of activated myofibroblast infiltration was used.  Cellular proliferation and microvascular density were assessed using nuclear stain Ki-67 antibody (Thermo Fisher Scientific, Waltham, Mass; dilution 1:500) and CD34 antibody (Bio-Rad, Hercules, Calif; dilution 1:100), respectively.  The slides were washed twice with TBST and once with TBS before incubating with (1) Goat anti-mouse HRP polymer secondary antibody (Abcam, ab127055) for SMA, CD68 and NK and (2) Goat anti-Rabbit HRP polymer secondary antibody (Abcam, ab214880) for CD34, Ki-67, CD80, NK, FGF-19 and VEGFR. After incubating with HRP polymer secondary antibodies for 90 minutes at room temperature the slides were washed twice with TBST and once with TBS before developing DAB (Diaminobenzidine) kit (Vector lab, SK-4105) and counterstained with hematoxylin. After dehydration steps, the slides were mounted with Permount.

The immunofluorescence antigen labeling of the paraffin-embedded rat hepatic tissue samples was performed using α-SMA antibody. The paraffin sections were de-paraffinized and treated with heat-mediated antigen retrieval using the sodium citrate buffer (pH 6). After three washes with TBS, the sections were incubated with 5% normal donkey serum (Jackson ImmunoResearch Lab Inc, West Grove PA) for an hour at room temperature.  Slides were then incubated with mouse anti-SMA (1:500, Abcam, ab 7817) overnight at 4^o^C. The slides were washed three times and incubated with Cy3-conjugated Donkey anti-mouse secondary antibody (Jackson ImmunoResearch Lab, 1:300). Samples were counterstained with Hoechst 33342 (Invitrogen) and washed three times with TBS. The slides were mounted with Prolong Gold anti-fade mounting media (Invitrogen).

Using velocity software, the fluorescent intensity of α-SMA positive cell per square area for of the imaged field for each specimen was determined to quantify the infiltrated periablational myofibroblast. The positive pixel intensity per mm square area for microvascular endothelial cell marker CD34, cell proliferative marker Ki-67, macrophages marker CD68, dendritic cells marker for CD80, natural killer cells marker KIR3DL1, fibroblast growth factor 19 FGF-19 and vascular endothelial growth factor receptor 1 VEGFR1 of the imaged field for each specimen was determined. The slides were microscopically analyzed by taking five random high-power fields for analysis of a minimum of three specimens for each parameter and scored in a blinded fashion to remove observer bias. α-SMA positive myofibroblasts in mouse tumor and liver tissue where quantified by means of percent cell positivity and rim thickness, respectively, under high field microscopy.

Serum quantification of HGF was performed using an ELISA kit (ab223862, Abcam). Briefly, liver/kidney samples were homogenized in RIPA lysis buffer containing 0.1% proteinase inhibitor (Sigma-Aldrich). The homogenates were then centrifuged at 14 000 rpm for 20 minutes at 4°C, and the total protein concentration was determined by using bicinchoninic acid. Following manufacturer recommendation, the HGF in the tissue periablational rim was determined and normalized again the total protein in the samples. All samples and standards were measured in triplicates, and the average value was recorded in picograms per milliliter.

*Statistical Analysis:* GraphPad Prism (version 7) coupled with excel spreadsheet was used for all statistical analysis with means ± standard deviations in graphical representation. Mean tumor size for the day of ablation (day 0) and day of sacrifice (day 3 or day 7) and immunohistochemical quantification were compared with analysis of variance, with testing including a posttreatment interaction term. Additional posthoc analysis was performed to evaluate the significance (≤0.05) of the various treatment groups. Linear regression analysis was used to determine the slopes (pre- and post-treatment) of tumor growth curves and the mean post-treatment growth curve slopes were calculated and compared by using analysis of variance and paired two-tailed t-tests.

**Supplementary Figure legends**

**
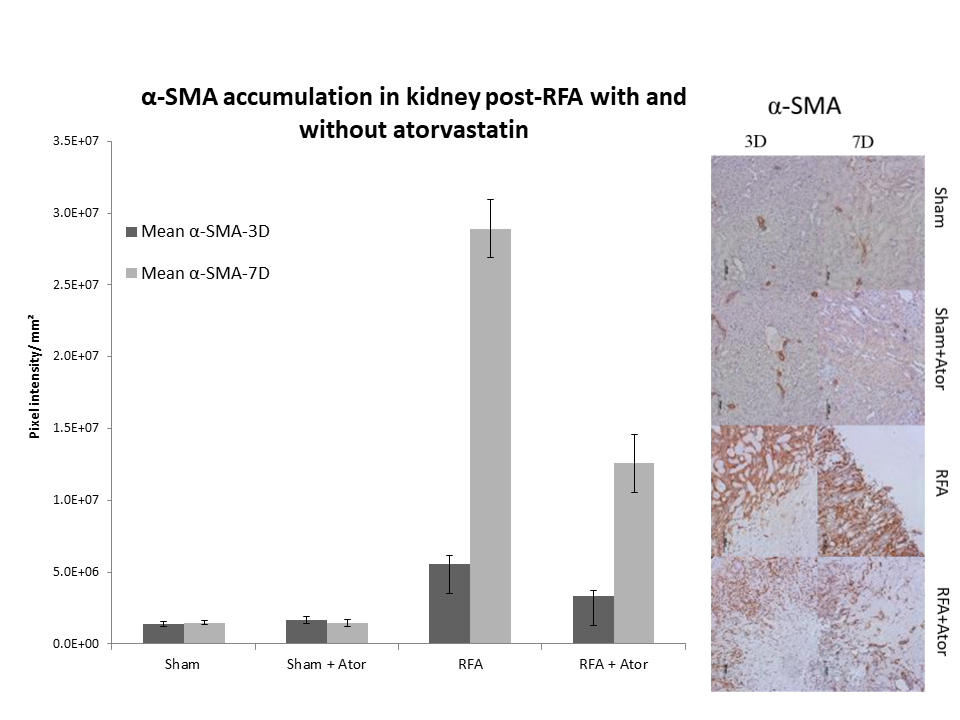
**

**Supplemental Figure 1. Atorvastatin partially suppresses RF induced periablational myofibroblast infiltration in ablated kidney.** Immunohistochemical analysis of myofibroblast infiltration at 3 and 7 days of renal periablational rim demonstrates statistically significant increased infiltration of activated myofibroblasts for RFA in comparison to control groups and RFA plus atorvastatin (p <0.05, all comparisons). Unlike liver models, RFA plus atorvastatin was statistically significantly greater than baseline levels of control groups (p <0.05, RF plus atorvastatin vs control groups, all comparisons) indicating only partial reduction of RF induced tumorigenesis.

**
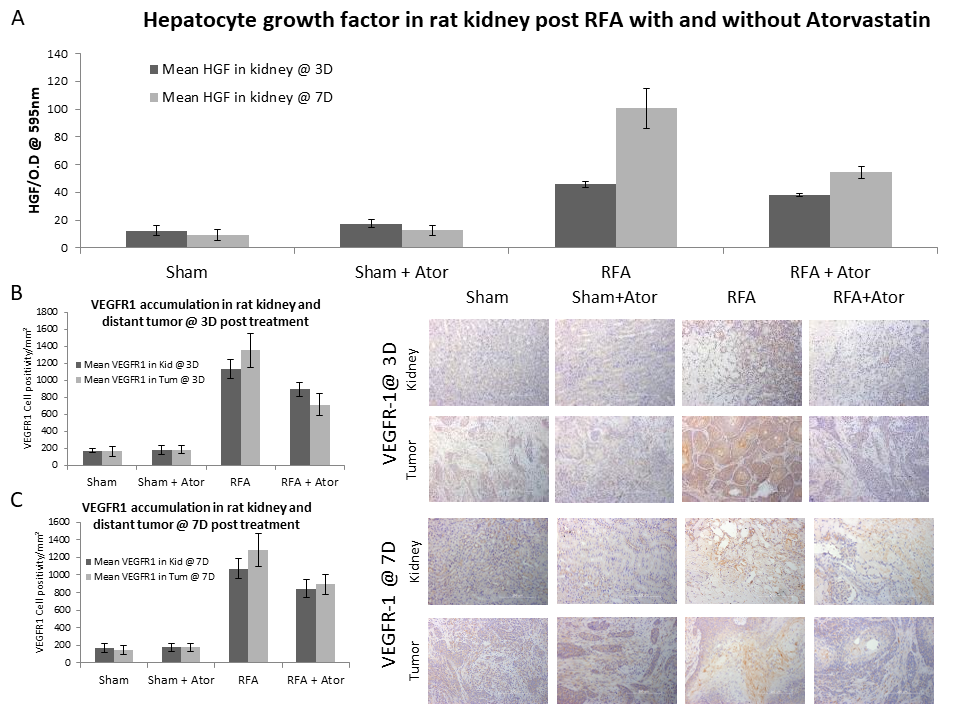
**

**Supplemental Figure 2. Myofibroblast blockade reduces renal RF driven tumor promoting cytokines.** Ablated kidney and distant tumor tissue were analyzed for established RFA-induced tumorigenic growth factors (HGF and VEGFR-1). ELISA (A) demonstrated significant upregulation of HGF in the RFA group compared to control groups (sham and sham plus atorvastatin treatment) at 3 and 7 days post-ablation. Increased HGF levels were reduced with atorvastatin compared to RFA alone treatment (p <0.05 all comparisons). Unlike liver, slightly higher HGF levels were demonstrated in RFA plus atorvastatin treatment in comparison to control groups (p <0.05 all comparisons). Immunohistochemical analysis (B&C) of VEGFR-1 demonstrated significantly increased FGF-19 (B) and VEGRFR-1 (C) levels in RFA renal and tumor tissues compared to sham or sham plus atorvastatin 7 day post-treatment (p-value <0.001, for all comparisons), which were successfully reduced in RFA plus atorvastatin (p-value <0.001, all comparisons to RFA and p-value <0.001, for comparisons with control groups).


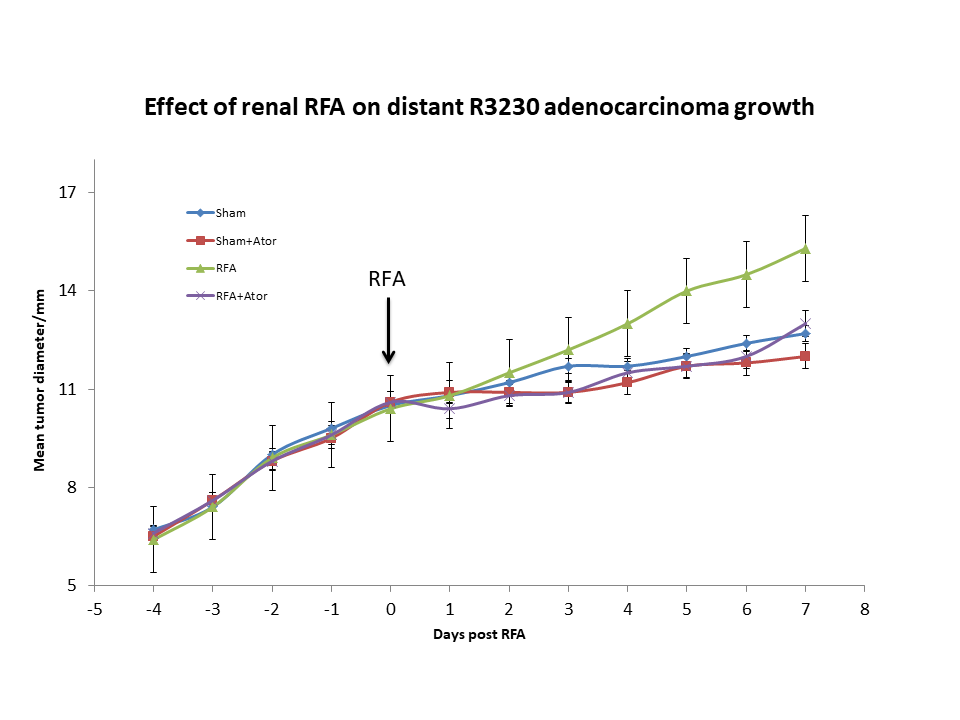


**Supplemental Figure 3. Myofibroblast blockade attenuates renal RFA-driven tumorigenesis of distant established tumors.** Curve demonstrates statistically significant increased mean tumor growth with kidney RFA as compared to sham, sham plus atorvastatin and RFA plus atorvastatin, starting 4 days after ablation (p <0.05, all comparisons). However, no statistically significant increase of tumor diameter was detected for RFA plus atorvastatin treatment in comparison to control groups (p-value >0.05, all comparisons).

**
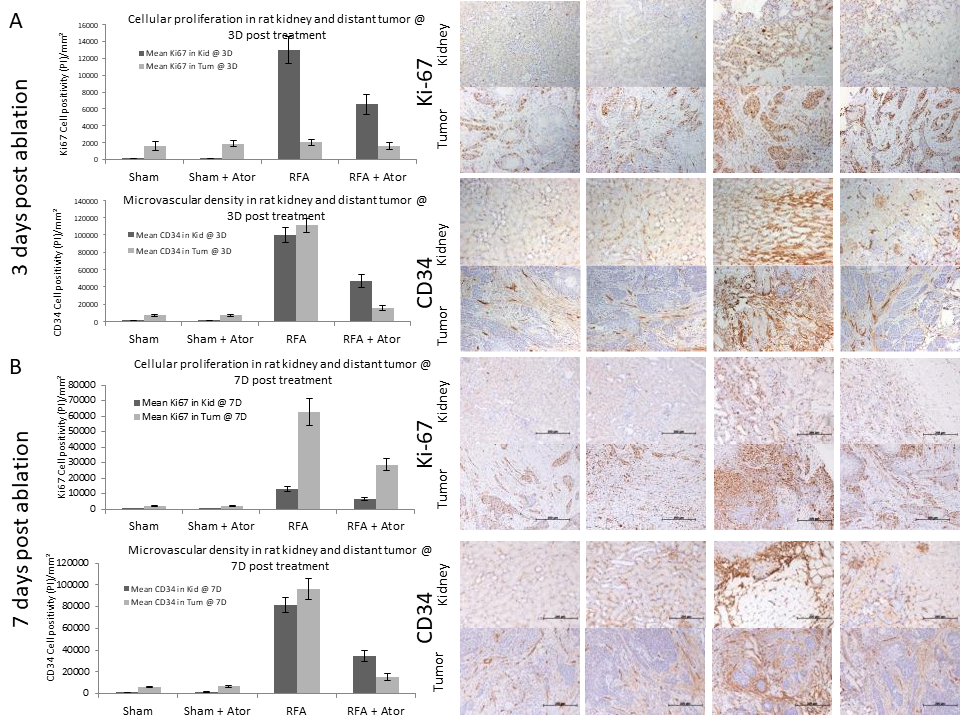
**

**Supplemental Figure 4. Myofibroblast blockade reduces post renal RF induced periablational and distant tumor proliferation.** Significantly increased Ki-67 and CD34 is noted in kidney periablational tissue and distant tumor nodules after RFA when compared to control groups and RFA plus atorvastatin treatment 3 days post-ablation (p <0.05, all comparisons). Slightly higher signal was demonstrated in RFA plus atorvastatin treatment in comparison to control groups (p <0.05, all comparisons). atorvastatin treatment partially reduced the increased signal uptake in kidney periablational rim and distant tumor after RFA (p <0.05, all comparisons)(A). Similar increased Ki-67 and CD34 signal were demonstrated in kidney periablational rim and distant tumor tissue after RFA in comparison to control groups and combined atorvastatin with RFA 7 days post-ablation (p <0.05, all comparisons). Partial attenuation was demonstrated with RFA plus atorvastatin treatment (p <0.05, all comparisons) (B).

**Supplementary Tables**

| Table 4: Effect of atorvastatin on cell trafficking in kidney periablational rim and, tumorigenic growth factors and proliferation in kidney periablational rim and distant macrometastasis after renal RF | | | | | | |
| --- | --- | --- | --- | --- | --- | --- |
|  | | Sham | Sham + Ator | RFA | RFA + Ator |  |
| ***Kidney Periablational rim*** | |  | | | |  |
| Hepatocyte Growth Factor | |  | | | |  |
| HGF  (OD±SD) | 3D | 12.4 ±3.6 | 27.4 ± 2.9 | 45.6 ±2.3 | 38.3 ±1.1 |  |
|  | 7D | 9.3 ± 3.8 | 12.5 ± 3.9 | 100.4 ± 14.3 | 54.4 ± 4.2 |  |
| Cellular infiltrates |  | | | | |  |
| α-SMA  ( PI/mm²±SD) | 3D | 1354451.2 ± 213685.3 | 1695067.3 ± 226102.0 | 5548831.2 ± 599395.7 | 3326989.7 ±407853.7 |  |
|  | 7D | 1487784.5 ± 167976.3 | 1461733.9 ± 246229.8 | 28917261.0 ± 2003874.2 | 12591947.0 ± 2015784.3 |  |
| CD68  ( PI/mm²±SD) | 3D | 98.5 ± 12.8 | 109.8 ± 12.7 | 2373.8 ± 321.3 | 1189.4 ± 169.2 |  |
|  | 7D | 103.6 ± 12.2 | 105.3 ± 11.1 | 2845.8 ± 223.4 | 1804.8 ± 194.1 |  |
| CD80  ( PI/mm²±SD) | 3D | 6.8 ± 1.4 | 6.7 ± 1.3 | 66.4 ± 10.2 | 20.4 ± 3.9 |  |
|  | 7D | 13.7 ± 2.3 | 13.9 ± 2.9 | 314.0 ± 37.5 | 55.1 ± 8.6 |  |
| Tumorigenic Growth factors |  | | | | |  |
| VEGFR-1  ( PI/mm²±SD) | 3D | 170.9 ± 25.4 | 176.9 ±48.0 | 1129.6 ± 109.5 | 892.6 ± 79.7 |  |
|  | 7D | 165.6 ± 54.2 | 174.7 ± 42.9 | 1068.2 ± 112.9 | 842.5 ± 100.1 |  |
| Proliferative indices |  | | | | |  |
| Ki-67  ( PI/mm²±SD | 3D | 87.1 ± 11.3 | 99.3 ± 12.9 | 12967.1 ± 1570.8 | 6534.6 ± 1157.1 |  |
|  | 7D | 93.8 ±14.0 | 97.1 ± 19.6 | 13010.8 ± 1518.1 | 6545.6 ± 1196.9 |  |
| CD34  ( PI/mm²±SD) | 3D | 933.1 ± 104.5 | 1052.3 ± 165.4 | 99522.7 ± 8876.7 | 46579.1 ± 7629.5 |  |
|  | 7D | 1005.0 ± 120.1 | 1232.1 ± 229.3 | 81062.5 ± 6937.2 | 34437.8 ± 4917.8 |  |
|  | | | | | |  |
| ***Distant adenocarcinoma/ R3230*** | |  | | | |  |
| Tumorigenic Growth factors | |  | | | |  |
| VEGFR-1  ( PI/mm²±SD) | 3D | 163.8 ± 58.2 | 180.0 ± 46.9 | 1350.2 ± 202.7 | 709.2 ± 128.4 |  |
|  | 7D | 148.4± 52.9 | 174.7 ± 46.8 | 1280.7 ± 189.1 | 890.1 ± 116.4 |  |
| Proliferative indices | |  | | | |  |
| Ki-67  ( PI/mm²±SD) | 3D | 1599.0 ± 484.6 | 1848.7 ± 373.5 | 2032.3 ± 333.8 | 28761.3 ± 4114.6 |  |
|  | 7D | 1843.9 ± 344.5 | 2032.2 ± 333.8 | 62728.2 ± 8802.2 | 28761.3 ± 4114.6 |  |
| CD34  ( PI/mm²±SD) | 3D | 6824.5 ± 711.9 | 6913.4 ± 955.4 | 111312.0 ± 8035.8 | 15973.8 ± 2888.6 |  |
|  | 7D | 6152.7 ± 522.6 | 6613.8 ± 916.2 | 96241.7 ± 9395.9 | 15089.6 ± 2951.2 |  |
